# A Simple, Sensitive, and Reliable Method for the Simultaneous Determination of Multiple Antibiotics in Vegetables through SPE-HPLC-MS/MS

**DOI:** 10.3390/molecules23081953

**Published:** 2018-08-06

**Authors:** Yao Feng, Wen-Juan Zhang, Yuan-Wang Liu, Jian-Ming Xue, Shu-Qing Zhang, Zhao-Jun Li

**Affiliations:** 1 Key Laboratory of Plant Nutrition and Fertilizer, Ministry of Agriculture, Institute of Agricultural Resources and Regional Planning, Chinese Academy of Agricultural Sciences, Beijing 100081, China; fengyao73@126.com (Y.F.); zhangwenjuan@lzb.ac.cn (W.-J.Z.); liuyuanwang199121@foxmail.com (Y.-W.L.); zhangshuqing@caas.cn (S.-Q.Z.); 2 China-New Zealand Joint Laboratory for Soil Molecular Ecology, Institute of Agricultural Resources and Regional Planning, Chinese Academy of Agricultural Sciences, Beijing 100081, China; 3 Beijing Key Laboratory of Detection and Control of Spoilage microorganisms and Pesticide Residues in Agricultural Products, Beijing University of Agriculture, Beijing 102206, China; 4 Scion, Private Bag 29237, Christchurch 8440, New Zealand; Jianming.Xue@scionresearch.com

**Keywords:** antibiotics, vegetable, SPE, HPLC-MS/MS

## Abstract

Antibiotics, widely used in livestock breeding, enter the environment through animal manure because of incomplete absorption in animals, especially the farmland ecosystem. Therefore, antibiotics may be adsorbed by plants and even become hazardous to human health through the food chain. In this study, a simple, sensitive, and reliable method was developed for the simultaneous determination of eleven antibiotics, including four sulfonamides, two tetracyclines, three fluoroquinolones, tylosin, and chloramphenicol in different vegetable samples using SPE-HPLC-MS/MS. Vegetable samples were extracted by acetonitrile added with hydrochloric acid (125:4, *v*/*v*). The extracts were enriched by circumrotating evaporation, and then cleaned through SPE on a hydrophilic-lipophilic balance (HLB) cartridge. All compounds were determined on a C_18_ reverse phase column through HPLC-MS/MS. The mean recoveries of 11 antibiotics from spiked samples of vegetables ranged from 71.4% to 104.0%. The limits of detection and quantification were 0.06–1.88 μg/kg and 0.20–6.25 μg/kg, respectively. The applicability of this technique demonstrated its good selectivity, high efficiency, and convenience by the analysis of 35 vegetable samples available from a vegetable greenhouse. Antibiotic residues in vegetables have aroused wide concern from the public. Therefore, standards should be established for antibiotic residues in vegetables to ensure food safety and human health.

## 1. Introduction

Antibiotics have been widely used to treat infectious diseases and promote growth for livestock and poultry [1,2]. It was estimated that about 14,600 tons of antibiotics were produced for animals in the United States in 2012 [3]. In China, 52% of all antibiotics (approximately 162,000 tons) were used for veterinary medicine in 2013 [4]. However, antibiotics can be weakly absorbed and incompletely metabolized in animal guts, and 30%–90% of administered antibiotics are excreted into the environment via feces and urine in an unchanged form [5]. The majority of excrements containing antibiotic residues have been frequently applied in agricultural fields at concentrations of μg/kg to mg/kg levels [6,7]. The persistence of antibiotics may have potential threats to the agro-ecological environment, especially vegetation growth and safety [8]. Existing studies have found that residual antibiotics in the soil have a great negative impact on the environmental non-target organisms, such as wheat and maize [9,10]. Furthermore, antibiotics can be taken up by various plants, crops, and soil animals. Residual antibiotics from the soils can be absorbed by vegetables on farmland as they grow, and even harm human health through the food chain [11,12]. There is evidence that antibiotics can perform biological accumulation and become distributed in the sequence leaf > stem > root [7]. Therefore, a wide concern has been aroused about vegetation safety and human health.

The characteristics of antibiotics are different because of their various chemical structures. In addition, multiple types of antibiotics are difficult to simultaneously analyze [13]. Although methods have been developed for the determination of multiple antibiotic residues in samples, most of them have focused on the detection of residues in environmental media such as soil, animal manures, sludge, and sewage. Additionally, some methods for food products have centered on matrices like milk, honey, and meat (Table 1). Vegetables have just become noticed following environmental media including soil and manure; in addition, the simultaneous extraction of antibiotic residues from vegetables is more difficult than from liquid food samples due to the presence of pigments such as chlorophyll, xanthophyll, and so on. Therefore, only a few methods have been described in the literature for the analysis of multiple antibiotics in vegetables. The analytical methods in vegetables were developed with detection limits in the range of 0.021–0.092 μg/kg and 0.575–1.538 μg/kg using UPLC-ESI-MS/MS and HPLC-FLD, respectively, but only for quinolone antibiotics [14,15]. Yu et al. [16] developed a QuEChERS-UHPLC-MS/MS for the determination of multiple antibiotics in leafy vegetables with average recoveries of only 57%–91% and limits of detection of 0.33–2.92 μg/kg. Comparing the existing methods [14,15,16,17,18], the present method was developed for the simultaneous determination of multiple classes of antibiotics with lower detection limits, which was highly sensitive and conveniently operated.

The objective of the present study is to develop a simple, sensitive, and reliable method to simultaneously determine eleven target antibiotics in various types of vegetables using solid phase extraction (SPE) with high-performance liquid chromatography tandem mass spectrometry (HPLC-MS/MS). The selected antibiotics include sulfonamides, tetracyclines, fluoroquinolones, tylosin, and chloramphenicol. Different proportions of extracting solvents for sample extraction and several SPE cartridges for clean-up were compared. The limits of detection (LODs), the limits of quantification (LOQs), recoveries, and linearity of the method were evaluated in detail. Finally, the method was applied to determinate 15 vegetable samples from a greenhouse and to validate the feasibility.

## 2. Results and Discussion

### 2.1. Validation of Extracting Agents

**Figure 1:**
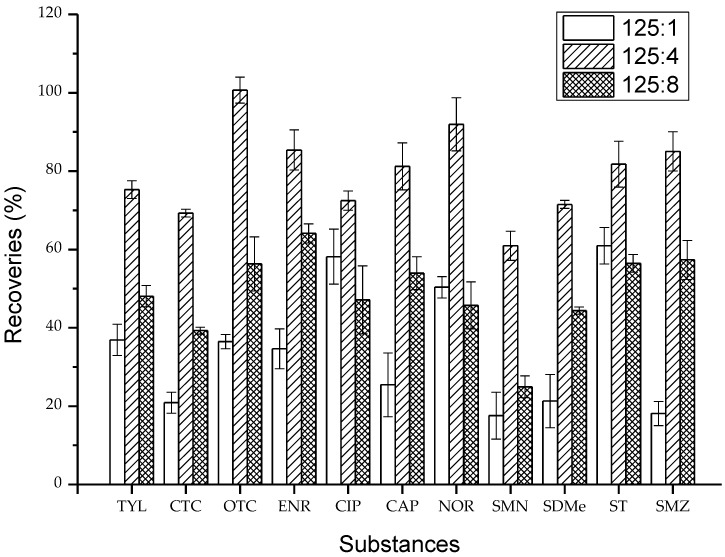
Comparison of 11 antibiotics recoveries from three different extraction methods for vegetables.

The pH is one of the dominant factors on varying physicochemical properties of multiple-class antibiotics. For example, when the pH is between pKa1 and pKa2, tetracyclines present a zwitterion (±0), while sulfanilamides are neutral molecules [17,29,30]. To obtain better and interference-less extracts, it is necessary to adjust the suitable solution pH value. Acetonitrile was reported to be a type of effective extract solution. During the extraction procedure, different proportions of the extraction solutions containing acetonitrile (ACN) and hydrochloric acid (HCl) with the volume ratio of 125:1 (M1), 125:4 (M2), and 125:8 (M3) were chosen as the extracting agents (Figure 1). Among the solvents, ACN/HCl (125:4, *v*/*v*) significantly promoted the recovery of analytes in vegetable samples. The recoveries of the target antibiotics from vegetables ranged from 60.9% to 100.7%, with relative standard deviations (RSDs) of between 1.0% and 6.8%. Extracting agents M1 and M3 gave lower recoveries with a range of 18.1%–58.2% and 24.9%–64.1%, with RSDs of 1.8%–8.2% and 0.9%–8.7%, respectively. ACN and HCl with the volume ratio of 125:4 could provide an appropriate acid base environment for the simultaneous extraction of multiple antibiotics, which can significantly improve the ionization efficiencies and recoveries of antibiotics.

The vegetable samples were determinated using SPE and HPLC-MS/MS after extraction by acetonitrile/hydrochloric acid (125:4, *v*/*v*), and the baseline of the chromatogram rose compared with the baseline of standard substances. This might be the result of antibiotic substances uniting with hydrochloric acid to form salts. Therefore, anhydrous sodium carbonate (Na_2_CO_3_) was added to the extracts for neutralizing superfluous HCl.

### 2.2. Optimization of SPE Cartridges

**Figure 2:**
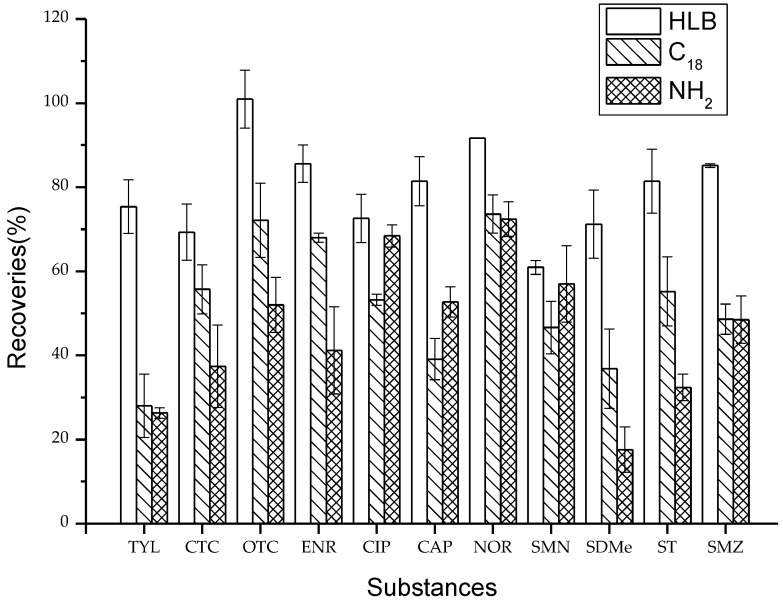
Comparison of 11 antibiotics recoveries from three different SPE cartridges for vegetables.

Selective adsorption is the basis for ensuring the efficient extraction of antibiotics from vegetable media. Three kinds of common SPE cartridges including Oasis HLB (Waters, Milford, MA, USA), C_18_ (Agela, Torrance, USA), and NH_2_ (Agela, Torrance, USA) were used to obtain efficient clean-up of the vegetable samples. The mean recoveries for the three SPE cartridges are shown in Figure 2. Both the C_18_ and NH_2_ cartridges had lower recoveries than the HLB cartridge. The recoveries ranged from 28.0% to 73.6% and from 17.6% to 72.4% for the C_18_ and NH_2_ cartridges, respectively, while those for the HLB cartridge ranged from 60.9% to 100.9%. The difference may be due to both the C_18_ and NH_2_ cartridges having high selectivity of the target compounds. The HLB cartridge contained lipotropic divinyl benzene and hydrophilic N-vinyl pyrrolidone as the adsorbents in contrast to the C_18_ and NH_2_ cartridges, which contained silica gel. The HLB cartridge has a higher adsorption capacity for antibiotics with a high polarity.

### 2.3. Method Efficiency

Each multicomponent standard contained every analyte at the same concentration. Aliquots of the 11 standard stock solutions were added to methanol to obtain 0.001, 0.005, 0.01, 0.05, 0.1, 0.5, 0.1, 0.5, 1, 5, and 10 μg/mL standard mixtures solutions to construct the calibration curves used for the quantification of target antibiotics in vegetables. All of targets showed good linearity over a range of 0.001–10 μg/mL, and the correlation coefficient (*r*^2^) of all calibration curves was >0.99. The limits of detection (LOD) and the limits of quantification (LOQ) were calculated as three times and ten times the standard deviation of the measurement of control samples divided by the slope of the calibration curve, respectively (Table 2). It is noted that the present method gave very low LOD and LOQ values ranging from 0.005–0.227 and 0.015–0.760 μg/kg. These data were substantially lower than those (0.33–1.73 and 1.10–5.77 μg/kg) obtained using QuEChERS and UHPLC-MS/MS analysis of the nineteen veterinary antibiotics in leafy vegetables [17]. Therefore, the method developed in the present study is recommended because of the high efficiency and low costs.

### 2.4. Method Precision and Accuracy

For testing the precision and accuracy of the method, aliquots of standard mixtures solutions were respectively spiked into the different vegetable samples including leek, celery, lentil, carob, and cauliflower to obtain 5, 10, and 50 μg/kg of spiked samples, and they were tested four times a day for three days following the protocol described in step 3.6. The obtained data were corrected and were quantified by the established calibration curves. The accuracy was expressed in terms of recovery rates and the precision was expressed as relative standard deviation (RSD). Table 3 shows that the recovery rates when the antibiotics were added at 5, 10, and 50 μg/kg were 71.9%–100.0%, 72.8%–99.2%, and 71.4%–104.0% respectively. Furthermore, the RSD of all analytes ranged from 1.2% to 13.4%. Different types of vegetables (leaf, root and stem, melon, and fruits) contained different vegetable fats, chlorophyll, and protein content. This would affect the extraction and purification of antibiotics, resulting in various recoveries of different vegetables. The results indicate that a surveillance programme for eleven veterinary compounds can be performed under the proposed chromatographic conditions. Therefore, this method can meet the requirements of different types of vegetables detection of antibiotics. According to the Decision of the European Commission 2002/657/EC [31] for the approval of a method for drug residue analysis, the average recovery of quantitative methods at analyte concentrations higher than 10 µg/kg should be 80%–110% and the RSD should not exceed 25.0%. The results presented in Table 3 indicate that all the analytes, except TYL, CIP, and CAP, meet these criteria.

### 2.5. Samples Analyses

Once the analytical methodology was validated, it was applied to detect the different types of vegetables. In total, 35 different vegetable samples was processed by the optimum procedures, as described in Section 3.6, with a blank sample to check and correct for possible contamination and interferences and a spiked blank at an intermediate concentration to calculate the extraction efficiency. Table 4 indicates that all target antibiotics were differently detected in the analyzed samples of scale farms. Tetracyclines were the predominant antibiotics in the different vegetables and the average residual concentration was 4.026 μg/kg. Fluoroquinolones, sulphonamides, CAP, and TYL contributed less residuals, with concentrations of 3.463, 0.123, 0.050, and 0.037 μg/kg, respectively. The results in this study are in accordance with the residual regulation of antibiotics in animal manures [20]. This is explained by the fact that the antibiotics caused bioaccumulation in vegetables due to the application of animal manures to farmland. Amongst the TCs, the detection frequencies were 71% for OTC with the highest mean residual concentration of 2.578 μg/kg, the maximum residual concentration was 4.706 μg/kg, and the minimum residual concentration was below LOD. CTC was detected in all the samples and the average concentration was 1.448 μg/kg, and the maximum and minimum concentration was 4.966 and 1.043 μg/kg, respectively. FQ was detected in 34 vegetable samples. However, three FQ antibiotics were not simultaneously detected and had different levels in the samples. The detection frequency of ENR was 54%, and the average residual concentration was 0.785 μg/kg. CIP was detected in 71% of samples, with the mean concentration of 0.785 μg/kg. NOR in FQ antibiotics had the highest detection frequency (86%) and the highest average concentration (1.743 μg/kg), indicating that there is likely to be widespread use of NOR in the livestock industry because of its cheapness. Similarly, SA was only undetected in one vegetable sample, while the detection frequencies were 66% for SMN, 51% for SDMe, 63% for ST, and 71% for SMZ. The residual concentrations of SAs ranged from ND to 1.956 μg/kg, and the average concentration was 0.123 μg/kg. The average concentrations of individual antibiotics decreased in the order of ST (0.083 μg/kg), SMN (0.023 μg/kg), SMZ (0.015 μg/kg), and SDMe (0.002 μg/kg). SAs and individual SA antibiotics were found at lower levels than the first two classes of antibiotics. CAP and TYL had a relatively low detection rate in 35 samples. The detection frequency of CAP was 28%, the average concentration was 0.050 μg/kg, and the maximum concentration was 0.698 μg/kg. TYL was detected in four samples, and the average concentration was 0.037 μg/kg.

## 3. Materials and Methods

### 3.1. Standards and Chemicals

The standards for 11 antibiotics (Supporting Information Table S1) including sulfamethazine (SDMe, 99.6%), sulfamethoxazole (SMZ, 99.5%), sulfathiazole (ST, 99.5%), sulfamonomethoxine (SMN, 95.0%), oxytetracycline (OTC, 96.5%), chlortetracycline (CTC, 93.0%), ciprofloxacin (CIP, 94.0%), norfloxacin (NOR, 99.1%), enrofloxacin (ENR, 99.5%), chloramphenicol (CAP, 98.6%), and tylosin (TYL, 98.0%) were obtained from Dr. Ehrenstorfer GmbH (Augsburg, Germany). HPLC-grade methanol (MeOH) and acetonitrile (ACN) were purchased from Fisher Scientific (Waltham, MA, USA). Anhydrous sodium carbonate (Na_2_CO_3_), hydrochloric acid (HCl), formic acid, acetic acid (HAc), and sodium hydroxide (NaOH) were of analytical reagent grade. Deionized water (DI) used in the experiments was prepared with a Milli-Q plus water system (Millipore, Billerica, MA, USA).

Each of the primary standards was accurately weighed (10 mg) into individual 10 mL amber volumetric flasks, dissolved, and made up to the mark with MeOH, in order to obtain individual stock standard solutions in MeOH with the concentration of 1 mg/mL, with the exception of fluoroquinolones (NOR, CIP, ENR, and NOR-D_5_) that were prepared in methanol with 0.03% NaOH added to enhance dissolution. To obtain working standard solutions, 0.01, 0.05, 0.1, 0.5, 1, 5, 10, 50, and 100 μL aliquots of the individual stock standard solutions were added into individual 10 mL brown volumetric flasks. All the solutions were stored at 4 °C.

### 3.2. Vegetable Samples

For method optimization, vegetable samples were collected from an ecological farm which did not use antibiotics in Shanxi province. For method proof, Chinese chives, celery, lentils, beans, and cauliflower were collected from a greenhouse in Shandong province in August 2015. All samples were put into dark plastic bags and kept in a cooler with ice until transported to the laboratory where all samples were stored at −80 °C before analysis.

### 3.3. Sample Preparation

During method validation and optimization, different proportions of extract agents and various SPE cartridges were tested for analysis of the target antibiotics in vegetables. To determine antibiotic recoveries, final concentrations of 5, 10, and 50 μg/kg in vegetables were obtained by adding 1.0 mL of mixed antibiotic methanol solution, with the above respective concentrations, to 1.0 g of lyophilized vegetable sample. Each sample was mixed well and placed in the fume hood at room temperature for 24 h for complete removal of the methanol by evaporation and for interaction of the analytes with the matrix in order to approximate real conditions. Each sample was extracted and analyzed in triplicate [32,33].

### 3.4. Sample Extraction and Clean-Up

**Figure 3:**
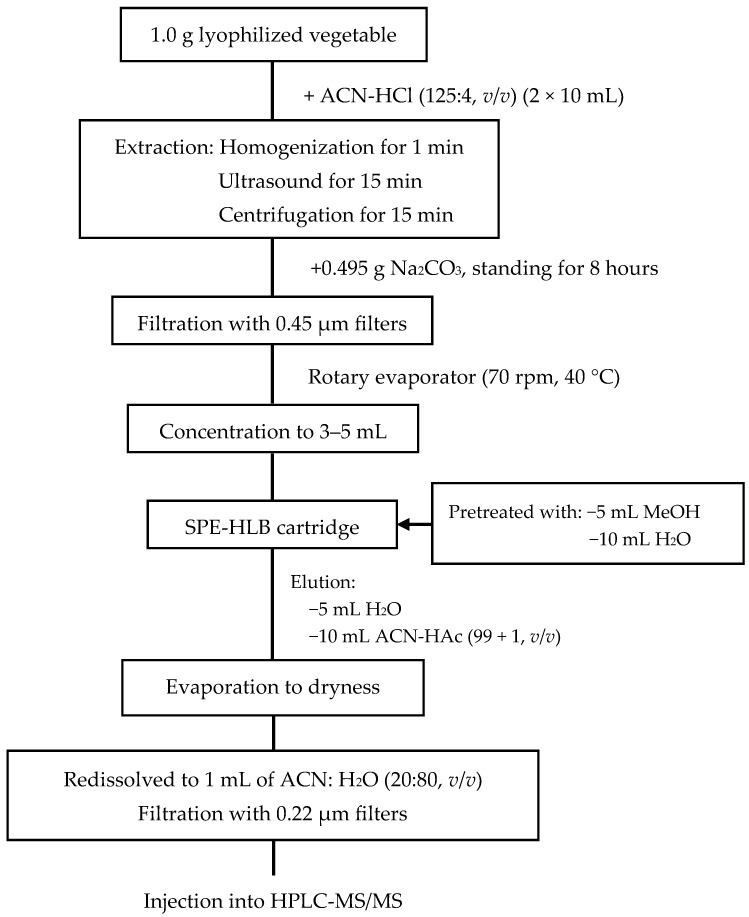
Procedure used for extraction of antibiotics. Optimized extraction, clean-up, and elution procedures developed in the present study are given.

In the present study, the vegetable samples were lyophilized using a vacuum freeze drier and sieved through a 2 mm sieve before further handling. The extracting agents were used and extraction was assisted with ultrasound. Clean-up steps were performed using solid-phase extraction. The extraction scheme used to extract the target compounds is illustrated in Figure 3.

### 3.5. HPLC-MS/MS Analysis

The analysis was performed using an HPLC-MS/MS system consisting of an Agilent 1200 high-performance liquid chromatograph (HPLC) system and an Agilent 6410 tandem triple-quadruple mass spectrometer (MS/MS) with an electrospray ionization (ESI) interface (Agilent Technologies, Santa Clara, CA, USA). The chromatographic separation was carried out with the use of a Waters Atlantis Sunfire C18 column (150 mm × 4.6 mm, 3.5 μm) at 35 °C. The flow rate was maintained at 0.3 mL/min, and the injection volume was 5 μL. The mobile phases A and B were 0.1% formic acid in water (A) and acetonitrile (B), respectively. The mobile phase gradient elution procedure took 28 min, as follows: 0–11 min, 80% A, 20% B; 11–16 min, 80%–40% A, 20%–60% B; 16–18 min, 40%–80% A, 60%–20% B; 18–28 min, 80% A, 20% B.

**Figure 4:**
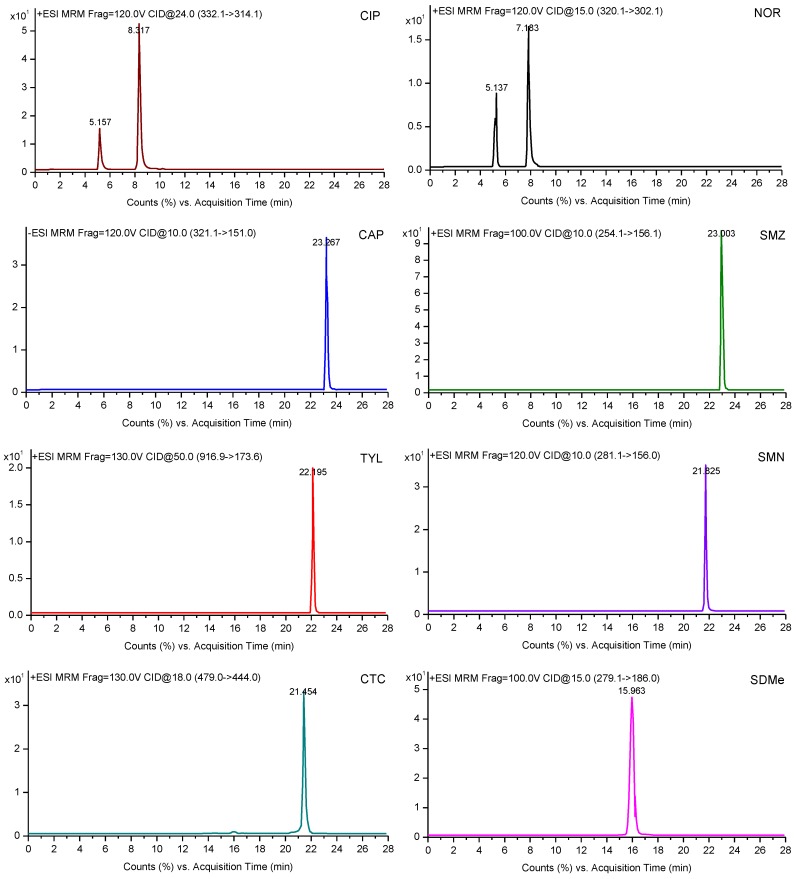

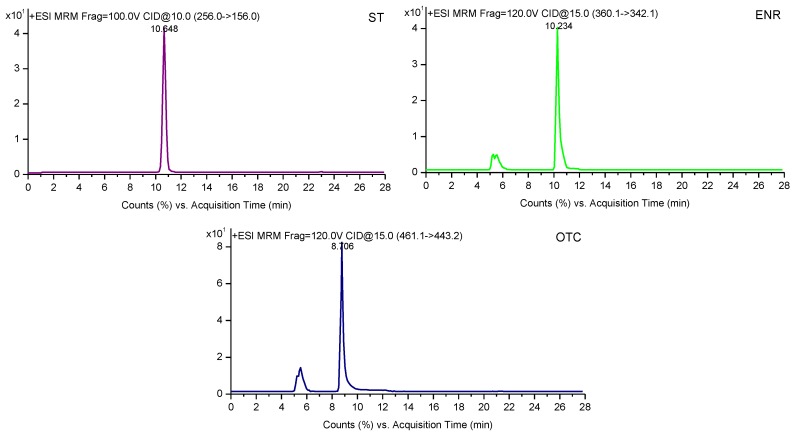
MS/MS spectrogram of eleven antibiotics.

For MS/MS detection, the instrument was operated in positive ion mode, with a capillary voltage of 3846 V, a drying gas temperature of 300 °C, and a drying gas flow rate of 10 L/min. Quantification of the selected substances was obtained using multiple reaction monitoring (MRM) detection. The MS/MS spectrogram and the monitored ions of the target analytes are shown in Figure 4 and Table 5, respectively. The chromatographic, interface, and MS/MS detector operating conditions are given in a detailed description in Supporting Information Table S2.

### 3.6. Detection of Antibiotics in Vegetables

**Figure 5:**
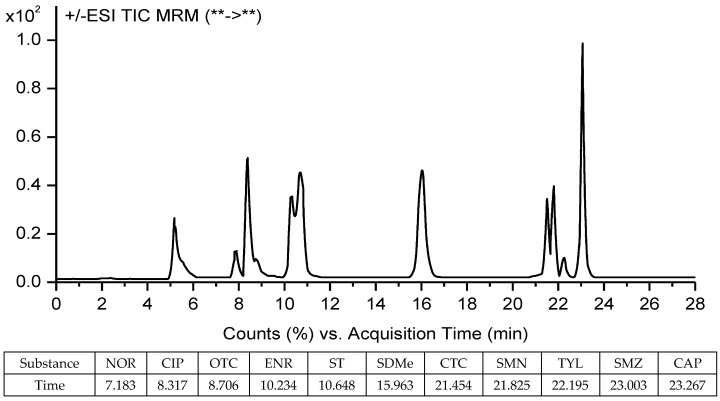
TIC chromatograms of a sample of extracted vegetable spiked (10 μg/g) with the selected 11 antibiotics.

A total of 1.0 g (±0.01 g) of vegetable sample was placed in a 50 mL centrifuge tube (Corning, New York, NY, USA) and 10 mL of acetonitrile/hydrochloric acid (125:4, *v*/*v*) was added to the tube. After vortexing (Berlin Wiggens, Berlin, Germany) the sample for 1 min, the mixture was sonicated (Ningbo Scientz, Ningbo, China) for 15 min at 4 °C and then centrifuged (Sartorius Sigma, St. Louis, MO, Germany) at 8000 *g* for 15 min at 4 °C. The supernatant was decanted into another 50 mL centrifuge bottle. The pellet was repeatedly extracted once with the same procedure using 10 mL of acetonitrile/hydrochloric acid (125:4, *v*/*v*), and the second supernatant was decanted into the same bottle. A total of 0.495 g of Na_2_CO_3_ was added to the supernatant for neutralizing superfluous HCl. After standing for 8 h, the extraction was centrifuged at 8000 *g* for 10 min at 4 °C and filtered through 0.22 μm of PVDF syringe filters (Tianjin Jinteng, Tianjin, China) into a 50 mL of round-bottom flask. The extract solution was concentrated to 3–5 mL on a rotary evaporator (70 rpm, 40 °C) (Hydrographic Guelph, Germany), and the liquid was purified and concentrated using an Oasis HLB (6 cm^3^, 500 mg) cartridge from Waters (Millford, MA, USA), which was preconditioned with 5 mL of methanol and 10 mL of DI water. The analyte was passed through the cartridge at a flow rate of 1 mL/min. After isolation, the cartridge was rinsed with 5 mL of DI water and dried under vacuum for 5 min. The analyte was eluted using 10 mL of ACN with HAc (99:1, *v*/*v*). The eluate was then evaporated to near dryness at 40 °C and redissolved in 1 mL of ACN with ultrapure water (20:80, *v*/*v*) for HPLC-MS/MS analysis (Figure 3). Where concentrations of antibiotics exceeded the chromatogram peak heights (Figure 5), samples were further diluted as required.

### 3.7. Statistical Analyses

Statistical significance tests were conducted using SPSS V.19 (IBM, Armonk, NY, USA). Graphs were generated with OriginPro 8.5 (OriginLab, Northampton, MA, USA) and Excel 2013 (Microsoft, Redmond, WA, USA).

## 4. Conclusions

The work presented in this paper shows a robust and viable method for the analysis of selected multi-class antibiotics including tetracyclines, fluoroquinolones, sulfonamides, tylosin, and chloramphenicol in different vegetable samples using high performance liquid chromatography-tandem mass spectrometry detection (HPLC-MS/MS). The method can be applied during the routine analysis conducted by laboratories. By analyzing the current method for determination, this paper puts forward the optimized method. The optimization of conditions for an instrument to establish and improve a method for the simultaneous detection of antibiotics residues in different vegetable samples provides a technical guarantee for the analysis of antibiotic residues in vegetables, in order to ensure the safety of every bite of food.

## Figures and Tables

**Table 1:** Comparison of existing determination methods for antibiotics residues in different environments.

Matrix Media	Methods	Number and Types of Antibiotics	Detection Limits	References
Vegetables	UPLC-ESI-MS/MS	4 (quinolones)	0.021–0.092 μg/kg	[14]
	HPLC-FLD	4 (quinolones)	0.575–1.538 μg/kg	[15]
	SPE-HPLC	6 (sulfonamides)	21.9–72.8 μg/kg	[16]
	UHPLC-MS/MS	20 (fluoroquinolones, sulfonamides and tetracyclines)	0.33–2.92 μg/kg	[17]
	LC-QqLIT-MS/MS	49 (sulfonamides, quinolones, macrolides, β-lactams and tetracyclines)	2–5 μg/kg	[18]
Soils	PLE-SPE-LC-MS/MS	5 (tetracyclines, macrolides and sulfonamides)	0.6–5.6 μg/kg	[19]
		8 (macrolides, ionophores and tiamulin)	0.2–1.6 μg/kg	[20]
Manure	LLE-SPE-LC-MS/MS	11 (tetracyclines, sulfonamides and tylosin)	2.7–32.1 μg/kg	[21]
	SPE-HPLC-MS/MS	3 (tetracyclines, quinolones and sulfadimidine)	0.04–0.25 mg/kg	[22]
	SPE-HPLC	11 (tetracyclines, quinolones, sulfonamides, tylosin and chloramphenicol)	0.1–1.9 μg/kg	[23]
Sewage sludge	USE-LC-MS/MS	10 (sulfonamides, macrolides, trimethoprim and chloramphenicol)	2.2–66.9 μg/kg	[24]
	SPE-LC-MS/MS	16 (fluoroquinolones, sulfonamides, trimethoprim, beta-lactams, nitroimidazoles and tetracyclines)	0.1–3.6 μg/L	[25]
Milk, honey and meat	SPE-UPLC-MS/MS	38 (beta-lactams, sulfonamides, quinolones, tetracyclines, macrolides and lincosamide)	0.1–5.0 μg/kg	[26]
	SPE-UHPLC QqTOF MS	104 (aminoglycosides, endectocides, fluoroquinolones, ionophores, β-lactams, macrolides, NSAIDs, phenicols, sulfonamides and tetracyclines)	—	[27]
	PLE-LC-MS/MS	7 (macrolides and lincosamides)	3–10 μg/kg (milk) 5–10 μg/kg (meat)	[28]

UPLC/UHPLC, ultra high-performance liquid chromatography; ESI, electrospray ionization; LC-MS/MS, liquid chromatography tandem mass spectrometry; HPLC, high-performance liquid chromatography; FLD, fluorimetric detector; SPE, solid phase extraction; QqLIT, quadrupole linear ion trap; PLE, pressurized liquid extraction; LLE, liquid-liquid extraction; USE, ultrasonic solvent extraction; MS, mass spectrometry; QqTOF, quadrupole time-of-flight.

**Table 2:** The LOD (μg/kg) and LOQ (μg/kg) of the selected veterinary antibiotics.

Substance	LOD (μg/kg)	LOQ (μg/kg)	Calibration Curve	Correlation Coefficient (*r*^2^)
TYL	0.005	0.017	y = 0.130x − 0.028	0.997
CTC	0.014	0.046	y = 0.049x + 0.100	0.998
OTC	0.227	0.760	y = 0.015x + 0.146	0.991
ENR	0.011	0.036	y = 0.025x + 0.064	0.991
CIP	0.026	0.088	y = 0.029x + 0.069	0.994
CAP	0.024	0.081	y = 0.454x − 0.112	0.999
NOR	0.138	0.459	y = 0.100x + 0.054	0.995
SMN	0.005	0.017	y = 0.051x − 0.079	0.994
SDMe	0.007	0.024	y = 0.015x − 0.076	0.993
ST	0.014	0.048	y = 0.022x − 0.030	0.999
SMZ	0.005	0.015	y = 0.022x − 0.033	0.999

Linear range: 0.001–10 μg/mL; y: peak area; x: mass concentration, μg/mL.

**Table 3:** Recovery of eleven antibiotics in vegetables (*n* = 5).

Substance	Spiked (μg/kg)	Leek		Celery		Lentil		Carob		Cauliflower	
		Recovery (%)	RSD (%)	Recovery (%)	RSD (%)	Recovery (%)	RSD (%)	Recovery (%)	RSD (%)	Recovery (%)	RSD (%)
TYL	5	89.2	4.0	81.6	3.1	81.4	6.7	81.1	7.6	79.2	3.5
	10	86.8	3.3	79.3	4.9	82.8	5.5	82.0	5.5	73.1	5.4
	50	93.2	3.5	78.4	6.8	87.4	5.4	89.7	4.6	71.4	7.3
CTC	5	91.4	3.9	82.2	4.6	92.7	4.3	92.8	4.5	97.1	3.5
	10	89.9	6.1	83.6	5.5	91.6	2.6	93.7	3.9	92.2	5.6
	50	91.3	4.7	86.9	7.3	94.3	6.5	94.0	5.2	89.4	7.2
OTC	5	76.2	4.7	91.8	4.2	91.1	4.7	93.2	7.7	91.9	4.3
	10	93.0	7.5	89.2	3.6	93.0	2.1	96.5	5.1	93.0	7.2
	50	96.4	3.7	94.1	7.1	94.6	1.9	93.3	3.2	89.9	4.3
ENR	5	100	4.6	90.3	7.7	98.3	4.2	92.9	2.5	93.2	3.2
	10	90.1	3.1	97.8	5.1	93.7	3.3	99.2	1.3	91.5	4.2
	50	95.0	6.8	100.5	9.1	97.5	4.5	95.3	4.4	97.9	3.8
CIP	5	87.2	7.9	82.8	6.0	81.6	1.9	71.9	3.1	73.5	2.4
	10	83.5	5.6	87.3	12.3	82.0	5.7	79.1	2.8	72.9	3.1
	50	85.9	5.2	104	13.4	89.2	2.8	88.0	3.3	78.3	4.0
NOR	5	77.3	7.6	96.1	6.1	86.5	5.9	88.0	2.6	91.4	4.4
	10	83.1	5.8	94.8	7.9	89.6	2.3	85.2	5.4	93.1	2.5
	50	85.6	4.6	96.7	8.9	91.3	1.7	87.5	1.7	90.4	3.3
SMN	5	86.6	3.7	86.1	7.3	87.4	5.6	95.7	3.6	86.2	2.5
	10	88.9	8.9	88.4	9.0	91.2	8.0	94.9	7.5	81.1	8.2
	50	89.4	5.6	89.8	2.5	95.1	4.5	97.2	4.1	89.4	5.3
SDMe	5	96.5	7.2	94.5	8.3	93.7	5.7	93.9	5.0	97.9	1.9
	10	91.4	5.0	96.2	5.9	91.8	1.2	96.2	2.4	95.7	3.2
	50	95.2	4.5	94.6	10.0	94.0	3.1	97.4	3.3	99.1	4.2
ST	5	81.5	6.2	80.2	9.6	89.2	4.7	94.4	4.0	91.8	4.9
	10	82.7	4.1	82.6	7.4	86.3	2.5	95.3	6.4	96.2	3.3
	50	83.1	3.0	87.9	3.9	88.6	6.4	97.1	4.3	93.8	3.2
SMZ	5	86.6	3.8	84.1	5.1	92.1	7.5	87.5	2.7	85.9	2.6
	10	88.9	4.9	87.4	6.8	97.5	8.0	84.9	1.9	88.7	1.5
	50	91.3	6.8	92.0	7.4	94.2	4.1	89.5	5.6	91.3	4.8
CAP	5	93.0	3.5	73.1	6.7	86.9	4.3	74.7	3.5	86.2	3.7
	10	92.4	6.8	77.7	5.5	92.3	2.9	80.6	5.4	72.8	5.2
	50	94.3	8.7	72.6	5.4	93.5	5.1	83.1	7.3	91.5	2.9

**Table 4:** Residues of 11 antibiotics in 35 vegetable samples (*n* = 5).

Substance	Freq ^1^ (%)	Residual Concentration (μg/kg)			
		Mean	Med.	Max	Min
OTC	71	2.578	3.463	4.706	ND ^2^
CTC	100	1.448	1.153	4.966	1.043
∑TCs ^3^	100	4.026	4.606	6.838	1.089
ENR	54	0.785	1.414	1.659	ND
CIP	71	0.935	1.302	1.414	ND
NOR	86	1.743	1.954	3.029	ND
∑QNs	97	3.463	3.336	5.251	ND
SMN	66	0.023	0.008	0.328	ND
SDMe	51	0.002	0.001	0.010	ND
ST	63	0.083	0.003	1.940	ND
SMZ	71	0.015	0.004	0.261	ND
∑SAs	97	0.123	0.023	1.956	ND
CAP	28	0.050	ND	0.698	ND
TYL	1	0.037	ND	0.425	ND

^1^ Freq.: frequency (%); Med.: median (μg/kg); Max: maximum (μg/kg); Min: maximum (μg/kg). ^2^ ND: not detected. ^3^ ΣTCs: total concentrations of two tetracyclines; ΣQNs: total concentrations of four fluoroquinolones; ΣSAs: total concentrations of four sulfonamides.

**Table 5:** Precursor masses and product ions for mass spectrometry MRM analysis of the selected antibiotics.

Substance	Parent Ion (*m*/*z*)	Quantitative Ion (*m*/*z*)	Collision Energy (eV)	Qualitative Ion (*m*/*z*)	Collision Energy (eV)	Fragment Voltage (V)
TYL	961.9	173.8	50	145.1	50	130
CTC	479.0	444.0	18	462.0	13	130
OTC	461.1	443.2	15	426.0	5	120
CAP	−321.1	151.0	10	257.0	5	120
SDMe	279.1	186.0	15	156.0	15	100
SMN	281.1	156.0	10	188.0	10	120
ST	256.0	156..0	10	108.0	10	100
SMZ	254.1	156.1	10	160.1	15	100
NOR	320.1	302.1	15	276.6	10	120
CIP	332.1	314.1	24	231.0	34	120
ENR	360.1	342.1	15	316.1	15	120

## Supplementary Materials

The following are available online, Table S1: The physicochemical property and primary usage of selected antibiotics, Table S2: LC and MS/MS operating conditions.
