# Supplementary material for: A Simple, Sensitive, and Reliable Method for the Simultaneous Determination of Multiple Antibiotics in Vegetables through SPE-HPLC-MS/MS
Source: Molecules. 2018 Aug 6;23(8):1953. doi: 10.3390/molecules23081953 (PMC6222851; doi:10.3390/molecules23081953)
Supplement: Supplementary file 1 [file molecules-23-01953-s001.pdf]

Table S1. The physicochemical property and primary usage of selected antibiotics

Classes	Substance	Acronym	p Ka	Log K_{ow}	Det. Purity	Molecular Structure
Macrolides (MAs)	Tylosin	TYL	7.1	3.5	98.9	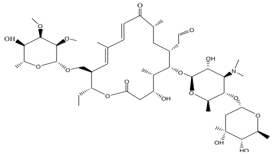
Tetracyclines (TCs)	Chlortetracycline	CTC	3.3/7.4/9.3	-0.62, -0.36	93.0	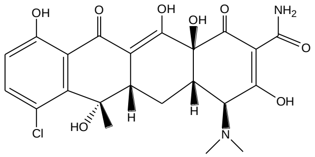
	Oxytetracycline	OTC	3.7/7.3/9.1	-0.90, -1.22	96.5	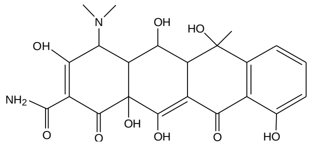
Chloramphenicols	Chloramphenicol	CAP	9.5	1.14	98.6	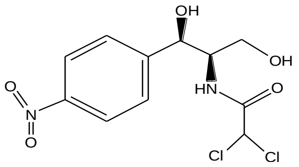
Sulfonamides (SAs)	sulfamethazine	SDMe	2.65/7.65	0.26	99.6	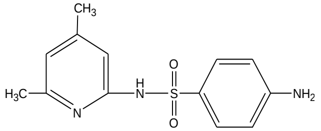
	Sulfamonomethoxine	SMN	6.05	0.18	95.0	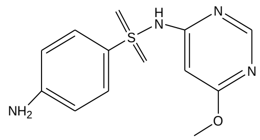

	Sulfathiazole	ST	7.10	0.02	99.5	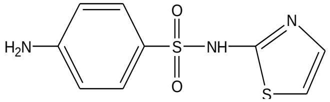
	Sulfamethoxazole	SMZ	1.4/5.8	0.89	99.5	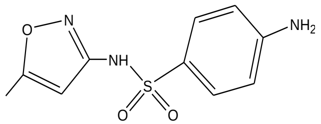
Fluoroquinolones (FQs)	Norfloxacin	NOR	6.22/8.51	-1.0, -1.7	99.1	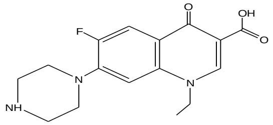
	Ciprofloxacin	CIP	6.43/8.49	0.28	94.0	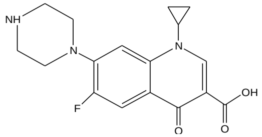
	Enrofloxacin	ENR	6.27/8.3	1.1	99.5	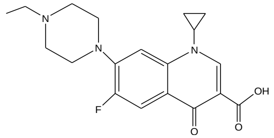

Table S2. LC and MS/MS operating conditions

LC-MS parameters	Value
Column	Waters Atlantis Sunfire C18 column (4.6 mm ×150 mm, 3.5 μm)
Flow	0.3 mL/min
Component A	0.1 % formic acid with H2O
Component B	ACN
Solvent program	0–11 min, 80% A, 20% B
	11–16 min, 80%-40% A, 20%-60% B
	16–18 min, 40–80% A, 60–20 % B
	18–28 min, 80% A, 20% B
MS method	MRM
MS mode	ESI-PI
Rough Vac	1.97E+0 Toor
High Vac	2.20E-5 Toor
Turbo1 Speed	100.0 %
MS1 Heater	100 °C
MS2 Heater	100 °C
Gas Temp	300 °C
Gas Flow	10.0 L/min
Nebulizer	20.0 psi
Capillary voltage	3846 V
Chamber Current	1.33 μA
Binary Pump: Ripple	-0.27 %
Capillary Current	11 nA
